# The linkage between the perception of neighbourhood and physical activity in Guangzhou, China: using street view imagery with deep learning techniques

**DOI:** 10.1186/s12942-019-0182-z

**Published:** 2019-07-25

**Authors:** Ruoyu Wang, Ye Liu, Yi Lu, Yuan Yuan, Jinbao Zhang, Penghua Liu, Yao Yao

**Affiliations:** 1 School of Geography and Planning, Sun Yat-Sen University, Xingang Xi Road, Guangzhou, 510275 China; 2 Guangdong Key Laboratory for Urbanization and Geo-Simulation, Sun Yat-Sen University, Xingang Xi Road, Guangzhou, 510275 China; 3 Department of Architecture and Civil Engineering, City University of Hong Kong, Hong Kong, SAR China; 4 School of Geography and Information Engineering, China University of Geosciences, Wuhan, 430074 China

**Keywords:** Physical activity (PA), Tencent Street View (TSV), Neighbourhood perception, Deep learning

## Abstract

**Background:**

Neighbourhood environment characteristics have been found to be associated with residents’ willingness to conduct physical activity (PA). Traditional methods to assess perceived neighbourhood environment characteristics are often subjective, costly, and time-consuming, and can be applied only on a small scale. Recent developments in deep learning algorithms and the recent availability of street view images enable researchers to assess multiple aspects of neighbourhood environment perceptions more efficiently on a large scale. This study aims to examine the relationship between each of six neighbourhood environment perceptual indicators—namely, wealthy, safe, lively, depressing, boring and beautiful—and residents’ time spent on PA in Guangzhou, China.

**Methods:**

A human–machine adversarial scoring system was developed to predict perceptions of neighbourhood environments based on Tencent Street View imagery and deep learning techniques. Image segmentation was conducted using a fully convolutional neural network (FCN-8s) and annotated ADE20k data. A human–machine adversarial scoring system was constructed based on a random forest model and image ratings by 30 volunteers. Multilevel linear regressions were used to examine the association between each of the six indicators and time spent on PA among 808 residents living in 35 neighbourhoods.

**Results:**

Total PA time was positively associated with the scores for “safe” [Coef. = 1.495, SE = 0.558], “lively” [1.635, 0.789] and “beautiful” [1.009, 0.404]. It was negatively associated with the scores for “depressing” [− 1.232, 0.588] and “boring” [− 1.227, 0.603]. No significant linkage was found between total PA time and the “wealthy” score. PA was further categorised into three intensity levels. More neighbourhood perceptual indicators were associated with higher intensity PA. The scores for “safe” and “depressing” were significantly related to all three intensity levels of PA.

**Conclusions:**

People living in perceived safe, lively and beautiful neighbourhoods were more likely to engage in PA, and people living in perceived boring and depressing neighbourhoods were less likely to engage in PA. Additionally, the relationship between neighbourhood perception and PA varies across different PA intensity levels. A combination of Tencent Street View imagery and deep learning techniques provides an accurate tool to automatically assess neighbourhood environment exposure for Chinese large cities.

## Background

Physical activity (PA) plays an important role in promoting health. Epidemiological evidence has suggested that performing regular PA decreases the risk of various chronic diseases, such as coronary calcification [1], diabetes [2], chronic nephrosis [3], cardiovascular disease [4], obesity [4] and various kinds of cancers [5]. PA also promotes mental health by improving subjective well-being [6] and reducing stress [7]. However, most Chinese people fail to meet the standard of national guidelines (150 min/week) and spend too much time on sedentary leisure activities (an average of 3 h/day) [8].

Until recently, most scholarly efforts have been made to identify individual and household factors that shape physical activity behaviours, including socio-economic status [9, 10], demographic features [11, 12] and genetic predisposition [13]. Scientific evidence is mounting that the neighbourhood built environment may influence PA behaviours [14–17]. Previous studies across different countries have found a strong linkage between objectively measured built environment features such as neighbourhood walkability [18], the presence of recreational facilities [19], mixed land use patterns [20] and the availability of green space [21, 22] and the intensity of PA. More recent studies have focused on the role of residents’ perceptions of the built environment and their engagement in PA [23–25]. Positive perceptions of the neighbourhood environment (e.g. feeling that a neighbourhood is safe and beautiful) may encourage residents to conduct PA [26–34]. By contrast, negative neighbourhood perceptions such as feeling bored and unsafe may decrease residents’ willingness to engage in PA [28, 31]. A possible explanation for these findings is that a pleasant and appealing environment arouses pleasant feelings, while a negative perception of the environment arouses unpleasant feelings. Thus, people may spend more leisure time in pleasing places rather than in unpleasing places [35–37].

Previous epidemiological studies have mainly used two approaches to assess different aspects of neighbourhood environments: questionnaires completed by participants [16, 30, 38–43] and systematic field observation conducted by trained auditors [44–46]. Questionnaire surveys, the most widely used method to assess respondents’ perception of the neighbourhood environment, often use Likert scales (e.g., rating the level of safety on a five-point scale) [28, 31, 47] or open-ended questions (e.g., “How do you feel about the neighbourhood?”) to assess respondents’ perceptions of their neighbourhood [48]. The largest drawback of the questionnaire survey approach is that it is time-consuming and costly to enrol participants, hire interviewers and carry out surveys. In addition, participants’ responses are subject to recall bias and social desirability bias. Another widely used approach is field observation [44–46]. However, in-person field observation is costly, time-consuming and limited in the number of sampled neighbourhoods, because trained auditors are hired to walk or drive around the neighbourhood in person and rate different aspects of the environment using auditing instruments.

Due to the drawbacks of questionnaire surveys and field observation, some scholars have started to use automated auditing techniques to assess neighbourhood environments [49, 50]. Google Street View (GSV) is a frequently used source of data for automated environmental assessment. The strength of using GSV data for health studies includes but is not limited to cost-effectiveness, time-effectiveness and similarity to humans’ perceptions of environments on the ground [21, 22]. Recent years have witnessed increasing interest in applying GSV data to investigate the linkage between neighbourhood characteristics (e.g. walkability, bikeability, street greenery and obesogenic features) and PA [49–56]. These studies have attempted to use street view data to assess objective features of the neighbourhood built environment, yet how people’s perceptions of these objective features influence their engagement in PA remains understudied. The recent progress in machine learning techniques and street-view imagery techniques has enabled researchers to predict human perceptions of the neighbourhood environment [57–59]. Machine learning techniques and other artificial intelligence (AI) methods offer a promising direction for environmental epidemiology and preventive health studies in metropolises. First, a metropolis usually covers a large area, and AI methods can assess neighbourhood environments on a large scale. Second, a neighbourhood environment may change substantially over time. AI methods, along with big data (i.e., GSV), can help policymakers to capture changes in real time. To the best of our knowledge, the technique of predicting human perceptions of the neighbourhood environment has not yet been used to study the association between neighbourhood environments and residents’ engagement in PA. To address the above research gap, this study aims to examine the relationships between six neighbourhood perceptual indicators—wealthy, safe, lively, depressing, boring and beautiful—and residents’ time spent on PA in Guangzhou, China. The six perceptual indicators are predicted using a combination of Tencent Street View (TSV) imagery and deep learning algorithms.

The following three hypotheses are put forward to direct our empirical studies: (1) positive perceptions of neighbourhoods are positively related to residents’ time spent on PA; (2) negative perceptions of neighbourhoods are negatively associated with residents’ time spent on PA; (3) the associations between neighbourhood perceptions and residents’ PA time vary depending on the intensity levels of PA. This study contributes to the literature by using novel automated methods to assess perceptions of neighbourhood environments and quantify their relationships with PA in Chinese cities.

## Method

### Study population

A questionnaire survey was conducted in Guangzhou between June and August 2016. The questionnaire was collected through home visit by 15 trained adult investigators. The target respondents were from the labour force population, so students and respondents under 18 years old were not interviewed. In the first step, 35 residential neighbourhoods (*she qu*) were randomly selected from six districts of Guangzhou (Liwan, Tianhe, Panyu, Baiyun, Haizhu, and Yuexiu) using a multi-stage stratified probability proportionate to population size (PPS) sampling approach. In the second step, we randomly selected sampled households from each of the 35 residential neighbourhoods using a systematic sampling method. Last, we selected one household member from each household using the Kish Grid method. The survey generated a total of 1029 valid participants. However, as some participants did not answer PA-related questions, the final sample size for this study was 808. Exact household addresses were not available due to privacy protection regulations, so it was not possible to generate respondent-specific buffers to assess environmental exposure.

### Outcome

The outcome variable in this study was leisure time and recreational outdoor PA time, which was assessed using the short form of the International Physical Activity Questionnaire (IPAQ) [60]. PA was categorised into three intensity levels: light PA, moderate PA and vigorous PA.

#### Light PA time

Respondents were asked “In the past 7 days, how many days did you go out for a walk (for relaxational and recreational purposes) for more than 10 min?” and “How many minutes on average did you spend on walking per day?” Light PA time was calculated by multiplying the number of days spent on walking by the average number of minutes spent on walking per day.

#### Moderate PA time

Respondents were asked “In the past 7 days, how many days did you participate in moderate PA (a brisk walk, dancing etc.) for more than 10 min?” and “How many minutes on average did you spend on moderate PA per day?” Moderate PA time was computed by multiplying the number of days spent on moderate PA by the average number of minutes spent on moderate PA per day.

#### Vigorous PA time

Respondents were asked “In the past 7 days, how many days did you participate in vigorous PA (aerobic fitness, running, fast cycling etc.) for more than 10 min?” and “How many minutes on average did you spend on vigorous PA per day?” Vigorous PA time was calculated by multiplying the number of days spent on vigorous PA by the average number of minutes spent on vigorous PA per day.

#### Total PA time

Total PA time was the sum of light, moderate and vigorous PA time.

### Human perceptions of neighbourhood appearance

We assessed neighbourhood perceptions of each neighbourhood based on street view images extracted from Tencent Map (https://map.qq.com), a web map service similar to Google Maps. Tencent Map provides a comprehensive service of streets view images, which can be retrieved with API [61]. We constructed sampling points 100 metres apart along the road network that was retrieved from OpenStreetMap [62]. For each sampling point, we took street view images in four headings (0°, 90°, 180°, and 270°) [61]. We obtained an average of 2105.9 images (SD = 768.0) from each neighbourhood.

A human–machine adversarial scoring system was developed to predict human perceptions of neighbourhood appearance from Tencent street view images. In the first step, we applied a semantic segmentation technique to accurately identify objects from street view images [63, 64]. A fully convolutional neural network (FCN-8s) was used to segment the street view images into more than 150 types of common objects (e.g., trees and grasses) [65, 66]. To train the network (FCN-8s), we used a collection of annotated images from the ADE20K scene parsing and segmentation database [65, 66].

In the second step, after obtaining the image segmentations, we employed a human–machine adversarial scoring system to measure six perceptual indicators of neighbourhood appearance (namely wealthy, safe, lively, beautiful, boring and depressing) following previous studies [58, 59]. We generated these six perceptual indicators only, because previous studies had used them to assess people’s general perceptions of their neighbourhood (including both positive and negative aspects of perception) [58, 59]. To obtain a training sample of neighbourhood perception, thirty volunteers (mean age = 35.68 years; 50% males) were invited to score a total of 3000 randomly chosen Tencent Street View images from 5 million street view photos of major Chinese cities (such as Beijing, Shenzhen, Guangzhou, Shanghai, Wuhan and Hangzhou) on these six attributes. Then, a random forest model for automatic rating was trained by fitting the inputted rated images, which were segmented into 150+ objects in the process of image segmentation [67]. We believe that the perceptions of these thirty volunteers can represent local residents’ perceptions of the neighbourhood environment for two reasons. First, many streetscapes in China’s largest cities are similar, due to the international guidelines on urban design and planning. Second, perceptions of a wealthy, safe, lively, beautiful, boring and depressing neighbourhood are similar between volunteers and local residents, who are similar in terms of cultural background and life experiences.

**Fig. 1:**
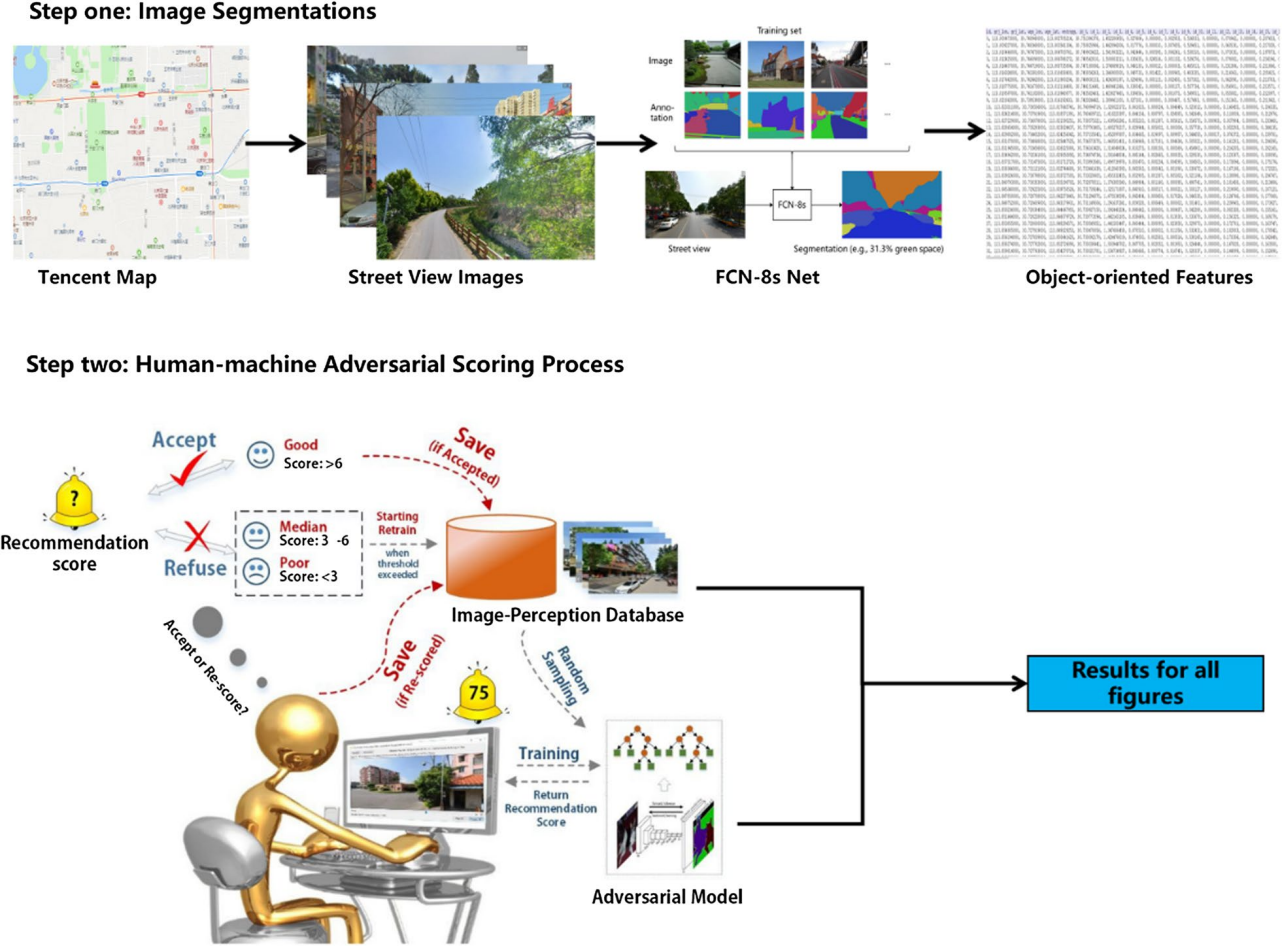
Workflow of predicting human perceptions of neighbourhood environments

We then applied a human–machine adversarial scoring approach to improve the accuracy of predictions of human perceptions [61]. Volunteers were asked to use a software tool that was connected to our trained scoring model. The trained scoring model automatically recommended a rating score for a new image and referred the image to volunteers, who calibrated the automatic scoring system by correcting the recommended score. The calibration process ended when the recommended scores provided by the model were close to scores given by volunteers (when the root-mean-square error score between the recommended scores and volunteers’ scores was below 5 for the last 100 images). Figure 1 summarizes the workflow of predicting human perceptions of neighbourhood environments.

Last, the accuracy and reliability were validated again. One hundred street view images were randomly selected, and perceptions of those images were again assessed by both our training model and five volunteers who were randomly chosen from all volunteers (mean age = 36.34 years; three males). The scores from the model were closely associated with volunteers’ scores regarding the six perceptual indicators: wealthy (Pearson correlation coefficient r = 0.98, p < 0.01), safe (r = 0.98, p < 0.05), lively (r = 0.97, p < 0.05), beautiful (r = 0.96, p < 0.05), boring (r = 0.96, p < 0.05) and depressing (r = 0.98, p < 0.05). This suggests that the predicted six indicators of neighbourhood appearance are reliable and valid, and that they can be used for preventive health studies.

Based on the automated scoring model, the six neighbourhood perceptual scores (wealthy, safe, lively, beautiful, boring and depressing) can be automatically generated for any street view image. We then calculated the perceptual score of each sampling point by averaging the corresponding scores of four images in different cardinal directions (0°, 90°, 180° and 270°). We calculated the perceptual score for each neighbourhood by averaging the corresponding scores of all sampling points within a 1 km circular buffer centred on the centroid of the residential neighbourhood (*she qu*).

### Covariates

We adjusted for a series of confounding covariates, including gender, age, marital status, education, household income, household size, length of stay in the neighbourhood, and the presence of functional restrictions (respondents were asked whether they had experienced any difficulty in functional ability in the past year).

### Analysis

We fitted multilevel linear regressions to assess the linkage between each aspect of human perceptions of neighbourhood appearance and PA [68]. Multilevel models were preferable to single-level models due to the hierarchical structure of our data. Variance inflation factors (VIF = 5.218) were used to investigate multicollinearity among variables. The intra-class correlation coefficient (ICC) for the null model (0.137) confirmed the necessity of using multilevel models, as between-neighbourhood variation accounted for 13.7 percent of the total variance in respondents’ PA time. We firstly regressed the outcome variable of total PA time on predictors (six neighbourhood perceptual indicators) and covariates (Model 1). Then, we estimated three models for sensitivity analysis (Models 2–4). First, people who have functional restrictions may have less PA than those who have no restrictions, regardless of the characteristics of their neighbourhoods. For this reason, we excluded respondents who reported functional restrictions from the sample and re-estimated the fully adjusted model (Model 2). Second, we excluded respondents aged above 70 from the sample and re-estimated the fully adjusted model (Model 3), because they were very unlikely to conduct vigorous PA, regardless of where they lived [12]. Third, we changed the radius of circular buffers from 1 to 1.5 km and re-ran the regressions (Model 4). Last, we categorised PA into three intensity levels (light, moderate and vigorous) (Models 5–7), given that the association between neighbourhood perception scores and PA was assumed to vary by the intensity levels of PA [69].

## Results

### Sample characteristics

Table 1 summarizes the characteristics of the study population. Results from the scored street view images showed that the mean value of the “wealthy” score across 35 neighbourhoods in Guangzhou was 4.902, the “safe” score was 4.718, the “lively” score was 4.832, the “depressing” score was 6.286, the “boring” score was 5.721 and the “beautiful” score was 3.884. High values of negative perception scores and low values of positive perception scores suggested that the sampled neighbourhoods had poor appearance. Results from the survey questionnaire showed that the average total PA of the 808 respondents was 198.741 min, with an SD of ± 206.255. Considering different intensity levels of PA, the average light PA time was 101.686 min, the average moderate PA time was 44.601 min, and the average vigorous PA time was 52.453 min. Overall, the mean age of respondents was 42.536 years, and 50.68% of them were female. About 2.843% of the respondents had only finished primary education, 51.298% had finished secondary education but not tertiary education, and 45.859% had a college degree or above. The average household size was 3.276, while the average length of living in the neighbourhood was 13.647 years. For household income, 8.034% of the respondents’ gross monthly household income was below 10,000 CNY, 70.952% was between 10,000 and 20,000 CNY, 15.204% was between 20,000 and 40,000 CNY, and 5.810% was above 40,000 CNY. There were 4.079% respondents who reported the presence of functional restrictions.

**Table 1:** Descriptive statistics for variables

Variables	Proportion/mean (SD) or %
Outcomes	
Total PA time (min)	198.7 (206.3)
Light PA time (min)	101.7 (73.0)
Moderate PA time (min)	44.6 (168.8)
Vigorous PA time (min)	52.5 (112.0)
Predictors	
Neighbourhood perception	
Wealthy (0–10)	4.9 (neighbourhood level: 0.4; point level: 0.4; image level: 0.3)
Safe (0–10)	4.7 (neighbourhood level: 0.4; point level: 0.4; image level: 0.4)
Lively (0–10)	4.8 (neighbourhood level: 0.4; point level: 0.4; image level: 0.4)
Depressing (0–10)	6.3 (neighbourhood level: 0.4; point level: 0.3; image level: 0.3)
Boring (0–10)	5.7 (neighbourhood level: 0.2; point level: 0.2; image level: 0.2)
Beautiful (0–10)	3.9 (neighbourhood level: 0.3; point level: 0.3; image level: 0.3)
Covariates	
Gender (%)	
Male	49.3
Female	50.7
Age (years)	42.5 (13.8)
Marital status (%)	
Single, divorced, and widowed	17.8
Married	82.2
Education (%)	
Primary school or below	2.8
High school	51.3
College and above	45.9
Household income (CNY/month)	
10,000 or below	8.0
10,000–20,000	71.0
20,000–40,000	15.2
40,000 or above	5.8
Household size (persons)	3.3 (0.9)
Length of staying in the neighbourhood (years)	13.6 (11.3)
Functional restriction (%)	
Yes	4.1
No	95.9

### The relationship between neighbourhood perception and PA

Table 2 shows the results of the associations between each aspect of neighbourhood perceptions and total PA time. We observed statistically significant and positive associations between positive neighbourhood perceptions and total PA time, as well as negative associations between negative neighbourhood perceptions and total PA time. Total PA time was positively associated with “safe” scores [Coef. = 1.495, SE = 0.558], “lively” scores [1.635, 0.789] and “beautiful” scores [1.009, 0.404], and was negatively associated with “depressing” scores [− 1.232, 0.588] and “boring” scores [− 1.227, 0.603]. Some of the covariates were also statistically significant. Males had more PA time than females [0.064, 0.028]. Age was positively correlated with total PA time [0.004, 0.002]. Compared with respondents with household income < 10,000 CNY, those with household income between 20,000 and 40,000 CNY had more total PA time [0.159, 0.069].

**Table 2:** The association between neighbourhood perception and total PA time

	Model 1
	Coef. (SE)
Fixed part	
Neighbourhood perception	
Wealthy	0.937 (1.024)
Safe	1.495*** (0.558)
Lively	1.635** (0.789)
Depressing	− 1.232** (0.588)
Boring	− 1.227** (0.603)
Beautiful	1.009** (0.404)
Male (ref: female)	0.064** (0.028)
Age	0.004*** (0.002)
Married (ref. = Single, divorced and widowed)	− 0.023 (0.047)
Education (ref: primary school or below)	
High school	− 0.049 (0.092)
College and above	− 0.022 (0.099)
Household income (ref: 10,000 CNY or below)	
10,000–20,000	0.104* (0.057)
20,000–40,000	0.159** (0.069)
40,000 or above	0.022 (0.079)
Household size	− 0.026 (0.019)
Length of staying in the neighbourhood	0.001 (0.002)
Functional restricted (ref: not restricted)	− 0.056 (0.074)
Constant	1.252 (6.487)
Random part	
Var (neighbourhoods)	0.022
Var (Individuals)	0.141
Number of individuals	808
Number of neighbourhoods	35
Log likelihood	− 375.623
AIC	791.246

*Coef.* coefficient, *SE* standard error

Significance levels: “*” p < 0.100, “**” p < 0.050, “***” p < 0.010

### Sensitivity analyses

Table 3 summarizes the results of robustness tests on the correlation between neighbourhood perception and total PA time. Despite some differences in magnitude, the neighbourhood perception-total PA time associations remained significant and the signs of their coefficients remained the same across all models.

**Table 3:** Robustness tests

	Model 2	Model 3	Model 4
	Coef. (SE)	Coef. (SE)	Coef. (SE)
Wealthy	0.854 (1.025)	0.966 (1.033)	1.189 (0.977)
Safety	1.343** (0.558)	1.483** (0.663)	1.585** (0.647)
Lively	1.445** (0.689)	1.372** (0.660)	1.688** (0.815)
Depressing	− 1.181** (0.523)	− 1.239** (0.528)	− 1.231** (0.608)
Boring	− 1.277** (0.608)	− 1.209** (0.601)	− 1.496** (0.747)
Beautiful	1.229** (0.497)	1.297** (0.481)	1.166** (0.499)
Number of observations	769	763	808

*Coef*. coefficient, *SE* standard error

Significance levels: “*” p < 0.100, “**”, p < 0.050, “***” p < 0.010. All models were adjusted for individual-level covariates. Model 2 excluded people who had functional restrictions, Model 3 excluded people aged > 70. Model 4 used a circular buffer with a radius of 1.5 km

### The relationship between neighbourhood perception and time spent on different intensity levels of PA

Table 4 summarizes the results of the association between neighbourhood perception and time spent on different intensity levels of PA. Model 5 showed that “safe” scores were positively associated with light PA time [Coef. = 2.183, SE = 1.104] while “depressing” scores were negatively associated with light PA time [− 2.202, 1.075]. Model 6 showed that “safe” scores [3.016, 1.454] and “beautiful” scores [2.227, 1.017] were positively associated with moderate PA time, while “depressing” scores were negatively associated with moderate PA time [− 2.712, 1.307]. Model 7 showed that “safe” scores [1.665, 0.751], “lively” scores [2.377, 1.624] and “beautiful” scores [1.734, 0.526] were positively associated with vigorous PA time, while “depressing” scores [− 1.148, 0.508] and “boring” scores [− 1.722, 0.858] were negatively associated with vigorous PA time.

**Table 4:** The association between neighbourhood perceptions and time spent on different intensity levels of PA

	Model 5	Model 6	Model 7
	Coef. (SE)	Coef. (SE)	Coef. (SE)
Wealthy	0.160 (2.043)	2.186 (2.563)	1.405 (2.108)
Safety	2.183** (1.104)	3.016** (1.454)	1.665** (0.751)
Lively	0.789 (1.104)	1.761 (1.977)	2.377** (1.624)
Depressing	− 2.202** (1.075)	− 2.712** (1.307)	− 1.148** (0.508)
Boring	− 0.079 (1.208)	− 0.911 (1.525)	− 1.722** (0.858)
Beautiful	0.378 (0.564)	2.227** (1.017)	1.734*** (0.526)
Number of observations	808	808	808

*Coef.* coefficient, *SE* standard error

Significance levels: “*” p < 0.100, “**”, p < 0.050, “***” p < 0.010. All models were adjusted for individual-level covariates. The outcome variables for Models 5, 6 and 7 were light PA time, moderate PA time and vigorous PA time, respectively

## Discussion

This study was among the first to examine the linkage between PA and human perceptions of neighbourhood appearance using street view images and deep learning techniques. Previous studies often use questionnaire surveys and field observation to assess neighbourhood perceptions and to find their associations with PA [16, 28, 38, 40–42, 44, 45]. We explored an innovative avenue to automatically assess perceptions of neighbourhood appearance with GSV and deep learning techniques. Our method is more accurate and can be more easily applied on a large scale than questionnaires and field observation [49].

This study demonstrates the potential of applying the combination of street view images and deep learning techniques to study the associations between perceived neighbourhood environments and PA. We found a strong correlation between neighbourhood perceptual indicators scored by volunteers and those scored automatically by machines (for all six indicators: r > 0.90, p < 0.05). Previous studies of 56 global cities have confirmed that evaluating the perception of the built environment using street view images and deep learning techniques can achieve high accuracy [58, 70]. Recent research conducted in Beijing, China also verified that residents’ perceptions of urban environments can be assessed accurately based on street view images and deep learning techniques [59]. Thus, street view images may become an important source for environment audit in future health studies, as web service providers such as Google and Tencent allow researchers to use their geo-tagged street view images.

Our findings that PA is associated with human perceptions of neighbourhood appearance in China complement existing evidence from developed countries [31–34] (e.g. Canada, Australia, the UK and the US). We found positive associations between positive neighbourhood perceptions (i.e. safety, lively and beautiful) and total PA time, and negative associations between negative neighbourhood perceptions (i.e. boring and depressing) and total time spent on PA. Other studies in developing countries are also in line with our results. For example, de Farias Júnior et al. [28] found that, in Brazil, neighbourhood positive perceptions such as safety increased people’s frequency and duration of PA. Positive neighbourhood perception in Nigeria was also reported to promote moderate-to-vigorous PA [49]. A systematic review focusing on Latin America proved that neighbourhood perception was associated with PA-related behaviours [16]. Positive neighbourhood perception may encourage residents to take PA by making the general neighbourhood environment attractive. On the other hand, negative neighbourhood perception may discourage PA through the same mechanism [36, 37]. In this study, all respondents have lived in their neighbourhood for at least 12 months, so they should be familiar with the perception of their neighbourhood environment, and this makes the neighbourhood perception even more important for their PA [71, 72].

Our findings further supported that the relationship between neighbourhood perception and time spent on PA varies across different intensity levels. Only the perceptions that neighbourhoods are safe and depressing influenced all three different intensity levels of PA, making them the most important neighbourhood perceptual indicators for PA behaviours. This finding corroborates the finding of Humpel et al. [17] that perceptions of safety were significantly correlated to light, moderate and vigorous PA in most of the 19 reviewed empirical studies. The sense of safety is a basic requirement for outdoor PA: people will instinctively reduce their usage of unsafe streets and other open spaces [40, 73, 74]. Another important finding is that with the increased intensity levels of PA, more neighbourhood perceptual indicators become significant. A possible explanation for this is that with the increase of intensity levels of PA [17], people may be more selective about the surrounding environment and hence have higher requirements for the environment’s quality. The indicators “lively”, “boring” and “beautiful” were associated with vigorous PA time. A possible explanation is that lively, interesting and beautiful neighbourhoods encourage residents to participate in legitimate routine activities (e.g. grocery shopping), thereby facilitating interaction among neighbours and promoting guardianship within the neighbourhood [75]. Both neighbourly interaction and surveillance on the streets will encourage residents to carry out vigorous PA.

Our findings have several planning and policy implications. First, we have demonstrated the possibility of using street view images and deep learning techniques to assess multiple aspects of neighbourhood environment perceptions, which may be related to residents’ willingness and ability to engage in PA. Urban planners and landscape designers can rely on automated assessment techniques to rate the quality of neighbourhood environments. Second, we have measured the association between different aspects of neighbourhood environments and time spent on PA, and our results can be used to identify neighbourhoods where PA is encouraged and those where it is discouraged. Planners and designers can encourage residents to engage in PA by improving neighbourhood outdoor environments (for example, enhancing the safety of the neighbourhood). Last, planners and designers are advised to pay attention to specific associations between specific intensity levels of PA and neighbourhood perceptions, because different intensity levels of PA are related to different aspects of environment perceptions.

Several limitations should be noted. First, the cross-sectional study design precludes any inference of causal relationships between neighbourhoods and residents’ PA. We cannot solve self-selection bias due to the cross-sectional research design. A natural experiment research design and cohort studies are advised for future studies. Second, time spent on PA was self-reported in this study, and the outcome variable may be prone to recall bias. Third, the present study used Tencent street view images, whereas most previous studies used Google street view images, which were unavailable in China. It would thus be difficult to compare the results from different data sources. Fourth, since we did not have respondents’ home addresses, we aggregated perceptual indicators at the neighbourhood level, which might lead to bias in the estimates of perception-PA relationships due to the ecological fallacy and Modifiable Area Unit Problem (MAUP) [76]. Also, we assumed that people living in the same neighbourhood might have the same environmental exposure, but people residing where two exposure buffers overlap may be exposed differently [77, 78]. Fifth, other aspects of neighbourhood environment perceptions require further investigation. In the current study, we focused only on six urban perceptual indicators (wealth, safety, liveliness, depression, boredom and beauty), because previous studies have validated their accuracy in assessing the actual conditions of neighbourhood environments [58, 59, 70]. Sixth, this study did not examine gender differences in the relationship between neighbourhood perceptions and PA (especially different levels of PA). Seventh, although some studies have indicated that participants of different ages and from different countries have similar perceptions of street view images, other studies have shown that gender [47] and place attachment [79] matter to the perception of street view images. Further research is needed examine the extent to which these factors affect the perception of street view images. Also, the missing data in this study may not occur randomly, which may cause bias in estimates. Ninth, the street view of Guangzhou might be different from images stored in the ADE20 K dataset, which compiles street view images of many countries. Tenth, some types of PA are not usually carried out on the streets (e.g. Taiji and basketball), and the perceived environment of streets could partially explain residents’ time spent on PA. Last, previous studies have indicated that the perceived environment may not match the objective environment [80–82], so perceptual biases associated with the measurement technique may still exist in this study.

## Conclusions

The present study examines the relationship between human perceptions of neighbourhood appearance and residents’ time spent on outdoor PA in Guangzhou, China. Results from multilevel regressions confirm that positive neighbourhood perceptions (safe, lively and beautiful) are positively associated with total PA time and that negative neighbourhood perceptions (boring and depressing) are negatively associated with total PA time. The perception of wealth is found to be unrelated to total PA time. The findings are justified by several robustness tests. Additionally, the relationship between neighbourhood perception and time spent on PA also varies across different intensity levels of PA. A combination of Tencent Street View imagery and deep learning techniques provides an accurate tool to automatically assess neighbourhood environment exposure for Chinese large cities.

## Data Availability

The datasets used and/or analysed during th e current study are available from the corresponding author on reasonable request.

## Abbreviations

PA: physical activity
Coef.: coefficient
SE: standard error
